# Supplementary material for: Personality moderates associations between personal time and parental well-being
Source: Commun Psychol. 2026 Jan 12;4:30. doi: 10.1038/s44271-026-00396-w (PMC12904863; doi:10.1038/s44271-026-00396-w)
Supplement: Supplementary file 2 — Supplementary Information [file 44271_2026_396_MOESM2_ESM.pdf]

Supplementary Information: Personality Moderates Associations Between Personal Time and Parental Well-being

Supplementary Table 1.

	Positive Affect					*Negative Affect*					Diurnal Cortisol Slope				
Predictors	*b*	*SE*	*β*	*standardized SE*	*p*	*b*	*SE*	*β*	*standardized SE*	*p*	*b*	*SE*	*β*	*standardized SE*	*p*
(Intercept)	2.35	0.12	0.01	0.04	**<0.001**	0.33	0.04	0.00	0.04	**<0.001**	-0.69	0.05	-0.00	0.05	**<0.001**
Personal time	0.12	0.03	0.05	0.01	**<0.001**	-0.05	0.02	-0.05	0.02	**0.001**	-0.07	0.03	-0.10	0.03	**0.013**
Neuroticism	-0.16	0.05	-0.14	0.05	**0.003**	0.11	0.02	0.27	0.04	**<0.001**	0.00	0.02	0.01	0.05	0.837
Extraversion	0.45	0.08	0.33	0.06	**<0.001**	-0.06	0.02	-0.13	0.05	**0.005**	-0.00	0.03	-0.00	0.06	0.942
Openness	-0.06	0.08	-0.03	0.05	0.499	0.07	0.02	0.12	0.04	**0.003**	-0.07	0.03	-0.12	0.05	**0.022**
Conscientiousness	0.29	0.08	0.17	0.05	**<0.001**	-0.04	0.02	-0.07	0.04	0.075	0.06	0.03	0.11	0.05	**0.021**
Agreeableness	-0.05	0.08	-0.03	0.05	0.561	0.05	0.02	0.09	0.04	**0.031**	0.07	0.03	0.14	0.05	**0.012**
Person mean personal time	0.18	0.15	0.05	0.04	0.211	-0.07	0.04	-0.06	0.04	0.081	0.08	0.05	0.07	0.05	0.129
Day in Study	-0.01	0.00	-0.03	0.01	**0.008**	-0.02	0.00	-0.13	0.02	**<0.001**	0.01	0.01	0.04	0.03	0.184

Personal time × Neuroticism	0.06	0.04	0.02	0.01	0.175	-0.06	0.02	-0.05	0.02	**0.006**	-0.09	0.04	-0.07	0.04	**0.050**
Personal time × Extraversion	-0.01	0.06	-0.00	0.01	0.884	0.04	0.03	0.03	0.02	0.235	-0.04	0.06	-0.03	0.05	0.491
Personal time × Openness	0.06	0.06	0.01	0.01	0.351	-0.07	0.03	-0.04	0.02	**0.035**	0.04	0.06	0.03	0.04	0.483
Personal time × Conscientiousness	0.05	0.06	0.01	0.01	0.480	-0.02	0.03	-0.01	0.02	0.504	0.01	0.06	0.01	0.04	0.819
Personal time × Agreeableness	-0.01	0.07	-0.00	0.01	0.865	-0.03	0.04	-0.02	0.02	0.453	0.06	0.07	0.04	0.05	0.387
**Random effects**															
Level-1 residual variance	0.17					0.05					0.05				
Random intercept variance	0.38					0.03					0.02				
Random slope variance (personal time)	0.02					0.00					0.02				
Correlation random intercept & slope	0.03					-0.71					0.33				
Marginal R^2 / Conditional R^2	0.183 / 0.754					0.110 / 0.421					0.059 / 0.363				

Note. All continuous predictors were grand-mean centered. Personal time was within-person centered. Marginal and conditional R^2 values represent the variance explained by fixed effects alone and by the full model, respectively. Bolded p values are significant at α = .05.

Supplementary Table 2.

	Diurnal Cortisol Slope				
Predictors	*Estimates*	*std. Error*	*std. Beta*	*standardized std. Error*	*p*
(Intercept)	-0.69	0.05	-0.00	0.05	**<0.001**
Personal time	-0.08	0.03	-0.10	0.03	**0.007**
Neuroticism	0.00	0.02	0.00	0.05	0.988
Person mean personal time	0.07	0.05	0.06	0.05	0.176
Day in Study	0.01	0.01	0.04	0.03	0.148
Personal time × Neuroticism	-0.10	0.04	-0.08	0.04	**0.022**
**Random effects**					
Level-1 residual variance	0.05				
Random intercept variance	0.02				
Random slope variance (personal time)	0.02				
Correlation random intercept & slope	0.33				
Marginal R^2 / Conditional R^2	0.022 / 0.358				

Note. All continuous predictors were grand-mean centered. Personal time was within-person centered. Marginal and conditional R^2 values represent the variance explained by fixed effects alone and by the full model, respectively. Bolded p values are significant at $\alpha = .05$

Supplementary Table 3.

	Positive Affect					Negative affect						Diurnal Cortisol Slope				
Predictors	Estimates	std. Error	std. Beta	standardized std. Error	p	Estimates	std. Error	std. Beta	standardized std. Error	p	std. p	Estimates	std. Error	std. Beta	standardized std. Error	p
(Intercept)	2.60	0.04	0.02	0.05	<0.001	0.17	0.01	0.00	0.03	<0.001	0.948	-0.66	0.03	-0.00	0.05	<0.001
leisure hrs	0.02	0.01	0.05	0.01	<0.001	-0.01	0.00	-0.04	0.02	0.031	0.031	-0.01	0.01	-0.08	0.04	0.034
leisure hrs person mean	-0.01	0.03	-0.02	0.05	0.647	0.01	0.01	0.04	0.03	0.248	0.248	0.00	0.01	0.00	0.05	0.992
studyday	-0.02	0.00	-0.05	0.01	<0.001	-0.01	0.00	-0.07	0.02	<0.001	<0.001	0.01	0.01	0.06	0.03	0.060
Stressor exposure	-0.19	0.02	-0.11	0.01	<0.001	0.16	0.01	0.27	0.02	<0.001	<0.001	0.04	0.02	0.07	0.04	0.056
Stressor exposure person mean	-0.54	0.15	-0.17	0.05	<0.001	0.23	0.04	0.20	0.03	<0.001	<0.001	-0.06	0.05	-0.05	0.05	0.279
**Random Effects**																
σ^2	0.16					0.05						0.05				

τ_{00}	0.46 $_{\text{MRID}}$	0.02 $_{\text{MRID}}$	0.02 $_{\text{MRID}}$
τ_{11}	0.00 $_{\text{MRID.leisure\_hrs\_within}}$	0.00 $_{\text{MRID.leisure\_hrs\_within}}$	0.00 $_{\text{MRID.leisure\_hrs\_within}}$
ρ_{01}	-0.28 $_{\text{MRID}}$	-0.57 $_{\text{MRID}}$	-0.03 $_{\text{MRID}}$
ICC	0.75	0.34	0.34
N	318 $_{\text{MRID}}$	318 $_{\text{MRID}}$	255 $_{\text{MRID}}$
Observations	2297	2297	788
Marginal R^2 / Conditional R^2	0.066 / 0.764	0.189 / 0.464	0.014 / 0.346

Supplementary Table 4.

	**Negative affect**				**Positive Affect**				**Diurnal Cortisol Slope**			
Predictors	*Estimates*	*std. Beta*	*p*	*std. p*	*Estimates*	*std. Beta*	*p*	*std. p*	*Estimates*	*std. Beta*	*p*	*std. p*
(Intercept)	0.16	0.00	**<0.001**	0.943	2.65	0.01	**<0.001**	0.782	-0.64	-0.00	**<0.001**	0.927
Personal time within	-0.04	-0.05	0.088	**0.006**	0.12	0.05	**0.002**	**<0.001**	-0.09	-0.10	**0.029**	**0.005**
Stressor exposure	0.16	0.27	**<0.001**	**<0.001**	-0.18	-0.11	**<0.001**	**<0.001**	0.04	0.07	0.069	0.069
stress pmean c	0.22	0.20	**<0.001**	**<0.001**	-0.55	-0.18	**<0.001**	**<0.001**	-0.05	-0.04	0.398	0.398
sex	-0.00	-0.00	0.907	0.907	-0.07	-0.04	0.363	0.363	-0.03	-0.05	0.310	0.310
Personal time pmean c	-0.03	-0.02	0.508	0.508	0.18	0.05	0.264	0.264	0.08	0.07	0.157	0.157
Day in study (0 = first day)	-0.01	-0.07	**<0.001**	**<0.001**	-0.02	-0.05	**<0.001**	**<0.001**	0.01	0.05	0.120	0.120
Personal time within × sex	-0.01	-0.01	0.673	0.673	-0.01	-0.00	0.811	0.811	0.02	0.01	0.790	0.790
**Random Effects**												
σ^2	0.05				0.16				0.05			
τ_{00}	0.02 $_{\text{MRID}}$				0.46 $_{\text{MRID}}$				0.03 $_{\text{MRID}}$			
τ_{11}	0.01 $_{\text{MRID.Personal time\_within}}$				0.01 $_{\text{MRID.Personal time\_within}}$				0.02 $_{\text{MRID.Personal time\_within}}$			
ρ_{01}	-0.65 $_{\text{MRID}}$				-0.01 $_{\text{MRID}}$				0.25 $_{\text{MRID}}$			
ICC	0.35				0.74				0.35			
N	318 $_{\text{MRID}}$				318 $_{\text{MRID}}$				255 $_{\text{MRID}}$			
Observations	2299				2299				788			
Marginal R^2 / Conditional R^2	0.187 / 0.471				0.073 / 0.761				0.021 / 0.365			

Supplementary Table 5.

	**Negative affect**				**Positive Affect**				**Diurnal Cortisol Slope**			
Predictors	*Estimates*	*std. Beta*	*p*	*std. p*	*Estimates*	*std. Beta*	*p*	*std. p*	*Estimates*	*std. Beta*	*p*	*std. p*
(Intercept)	0.16	0.00	**<0.001**	0.941	2.60	0.01	**<0.001**	0.772	-0.65	-0.00	**<0.001**	0.929
Personal time within	-0.04	-0.05	0.059	**0.005**	0.14	0.05	**0.001**	**<0.001**	-0.08	-0.10	0.075	**0.005**
Stressor exposure	0.16	0.27	**<0.001**	**<0.001**	-0.18	-0.11	**<0.001**	**<0.001**	0.04	0.07	0.073	0.073
stress pmean c	0.22	0.20	**<0.001**	**<0.001**	-0.55	-0.17	**<0.001**	**<0.001**	-0.04	-0.04	0.459	0.459
childinhh numb c	-0.00	-0.00	0.899	0.899	0.01	0.01	0.760	0.760	0.00	0.00	0.960	0.960
Personal time pmean c	-0.03	-0.02	0.517	0.517	0.18	0.05	0.246	0.246	0.08	0.07	0.161	0.161
Day in study (0 = first day)	-0.01	-0.07	**<0.001**	**<0.001**	-0.02	-0.05	**<0.001**	**<0.001**	0.01	0.05	0.126	0.126
Personal time within × childinhh numb c	0.00	0.00	0.873	0.873	-0.02	-0.01	0.470	0.470	0.00	0.00	0.903	0.903
**Random Effects**												
σ^2	0.05				0.16				0.05			
τ_{00}	0.02 $_{MRID}$				0.46 $_{MRID}$				0.03 $_{MRID}$			
τ_{11}	0.01 $_{MRID.Personal\ time\_within}$				0.01 $_{MRID.Personal\ time\_within}$				0.02 $_{MRID.Personal\ time\_within}$			
ρ_{01}	-0.66 $_{MRID}$				-0.01 $_{MRID}$				0.23 $_{MRID}$			
ICC	0.35				0.74				0.35			
N	317 $_{MRID}$				317 $_{MRID}$				254 $_{MRID}$			
Observations	2292				2292				786			
Marginal R^2 / Conditional R^2	0.187 / 0.471				0.071 / 0.762				0.019 / 0.366			

Supplementary Table 6.

	Negative affect				Positive Affect				Diurnal Cortisol Slope			
Predictors	*Estimates*	*std. Beta*	*p*	*std. p*	*Estimates*	*std. Beta*	*p*	*std. p*	*Estimates*	*std. Beta*	*p*	*std. p*
(Intercept)	0.16	0.00	**<0.001**	0.945	2.62	0.01	**<0.001**	0.781	-0.65	-0.00	**<0.001**	0.988
Personal time within	-0.04	-0.04	**0.005**	**0.008**	0.11	0.05	**<0.001**	**<0.001**	-0.08	-0.09	**0.006**	**0.009**
Stressor exposure	0.16	0.27	**<0.001**	**<0.001**	-0.18	-0.11	**<0.001**	**<0.001**	0.03	0.06	0.093	0.093
stress pmean c	0.22	0.20	**<0.001**	**<0.001**	-0.54	-0.17	**<0.001**	**<0.001**	-0.03	-0.03	0.569	0.569
min agechild c	-0.00	-0.01	0.766	0.766	-0.01	-0.03	0.479	0.479	0.01	0.12	**0.010**	**0.010**
Personal time pmean c	-0.03	-0.02	0.536	0.536	0.20	0.06	0.214	0.214	0.05	0.05	0.329	0.329
Day in study (0 = first day)	-0.01	-0.07	**<0.001**	**<0.001**	-0.02	-0.05	**<0.001**	**<0.001**	0.01	0.05	0.117	0.117
Personal time within × min agechild c	0.00	0.02	0.309	0.309	0.00	0.00	0.901	0.901	0.01	0.04	0.241	0.241
**Random Effects**												
σ^2	0.05				0.16				0.05			
τ_{00}	0.02 MRID				0.46 MRID				0.02 MRID			
τ_{11}	0.01 MRID.Personal time_within				0.01 MRID.Personal time_within				0.02 MRID.Personal time_within			
ρ_{01}	-0.65 MRID				-0.01 MRID				0.20 MRID			
ICC	0.35				0.74				0.34			
N	318 MRID				318 MRID				255 MRID			
Observations	2299				2299				788			
Marginal R^2 / Conditional R^2	0.188 / 0.471				0.071 / 0.761				0.035 / 0.364			

Supplementary Table 7.

	Negative affect				Positive Affect				Diurnal Cortisol Slope			
Predictors	*Estimates*	*std. Beta*	*p*	*std. p*	*Estimates*	*std. Beta*	*p*	*std. p*	*Estimates*	*std. Beta*	*p*	*std. p*
(Intercept)	0.18	0.00	**<0.001**	0.924	2.54	0.01	**<0.001**	0.813	-0.61	-0.00	**<0.001**	0.967
Personal time within	-0.02	-0.04	0.399	**0.007**	0.09	0.04	0.063	**<0.001**	-0.02	-0.09	0.678	**0.010**
Stressor exposure	0.16	0.27	**<0.001**	**<0.001**	-0.18	-0.11	**<0.001**	**<0.001**	0.03	0.06	0.095	0.095
stress pmean c	0.22	0.20	**<0.001**	**<0.001**	-0.54	-0.17	**<0.001**	**<0.001**	-0.04	-0.03	0.504	0.504
age c	0.00	0.03	0.307	0.307	-0.01	-0.07	0.152	0.152	0.00	0.10	**0.041**	**0.041**
Personal time pmean c	-0.03	-0.03	0.399	0.399	0.22	0.07	0.168	0.168	0.05	0.05	0.345	0.345
Day in study (0 = first day)	-0.01	-0.07	**<0.001**	**<0.001**	-0.02	-0.05	**<0.001**	**<0.001**	0.01	0.05	0.126	0.126
Personal time within × age c	0.00	0.01	0.409	0.409	-0.00	-0.01	0.616	0.616	0.01	0.05	0.209	0.209
**Random Effects**												
σ^2	0.05				0.16				0.05			
τ_{00}	0.02 MRID				0.46 MRID				0.02 MRID			
τ_{11}	0.01 MRID.Personal time_within				0.01 MRID.Personal time_within				0.02 MRID.Personal time_within			
ρ_{01}	-0.66 MRID				-0.02 MRID				0.20 MRID			
ICC	0.35				0.74				0.35			
N	318 MRID				318 MRID				255 MRID			
Observations	2299				2299				788			
Marginal R^2 / Conditional R^2	0.189 / 0.471				0.075 / 0.761				0.029 / 0.367			

Supplementary Table 8.

	Negative affect					
Predictors	*Estimates*	*std. Error*	*std. Beta*	*standardized std. Error*	*p*	*std. p*
(Intercept)	0.27	0.02	0.01	0.04	**<0.001**	0.819
timeoneself within	-0.05	0.02	-0.06	0.02	**0.037**	**0.001**
neuro c	0.11	0.02	0.27	0.04	**<0.001**	**<0.001**
sex	-0.01	0.02	-0.02	0.04	0.555	0.604
open c	0.05	0.03	0.08	0.04	0.064	**0.028**
timeoneself pmean c	-0.08	0.04	-0.07	0.04	0.060	0.060
studyday	-0.02	0.00	-0.13	0.02	**<0.001**	**<0.001**
timeoneself within × neuro c	-0.02	0.03	-0.05	0.02	0.608	**0.010**
timeoneself within × sex	-0.01	0.03	-0.01	0.02	0.721	0.536
neuro c × sex	0.01	0.03	0.02	0.04	0.690	0.690
timeoneself within × open c	-0.07	0.04	-0.03	0.02	0.082	**0.042**
sex × open c	-0.01	0.04	-0.01	0.04	0.833	0.833
(timeoneself within × neuro c) × sex	-0.09	0.05	-0.04	0.02	**0.046**	**0.046**
(timeoneself within × sex) × open c	0.01	0.06	0.00	0.02	0.867	0.867

**Random Effects**	
σ^2	0.05
$\tau_{00\ MRID}$	0.03
$\tau_{11\ MRID.timeoneself\_within}$	0.00
$\rho_{01\ MRID}$	-0.76
ICC	0.36
N_{MRID}	318
Observations	2299
Marginal R^2 / Conditional R^2	0.097 / 0.422

Supplementary Figure 1. Interaction between gender, personal time, and neuroticism on negative affect.

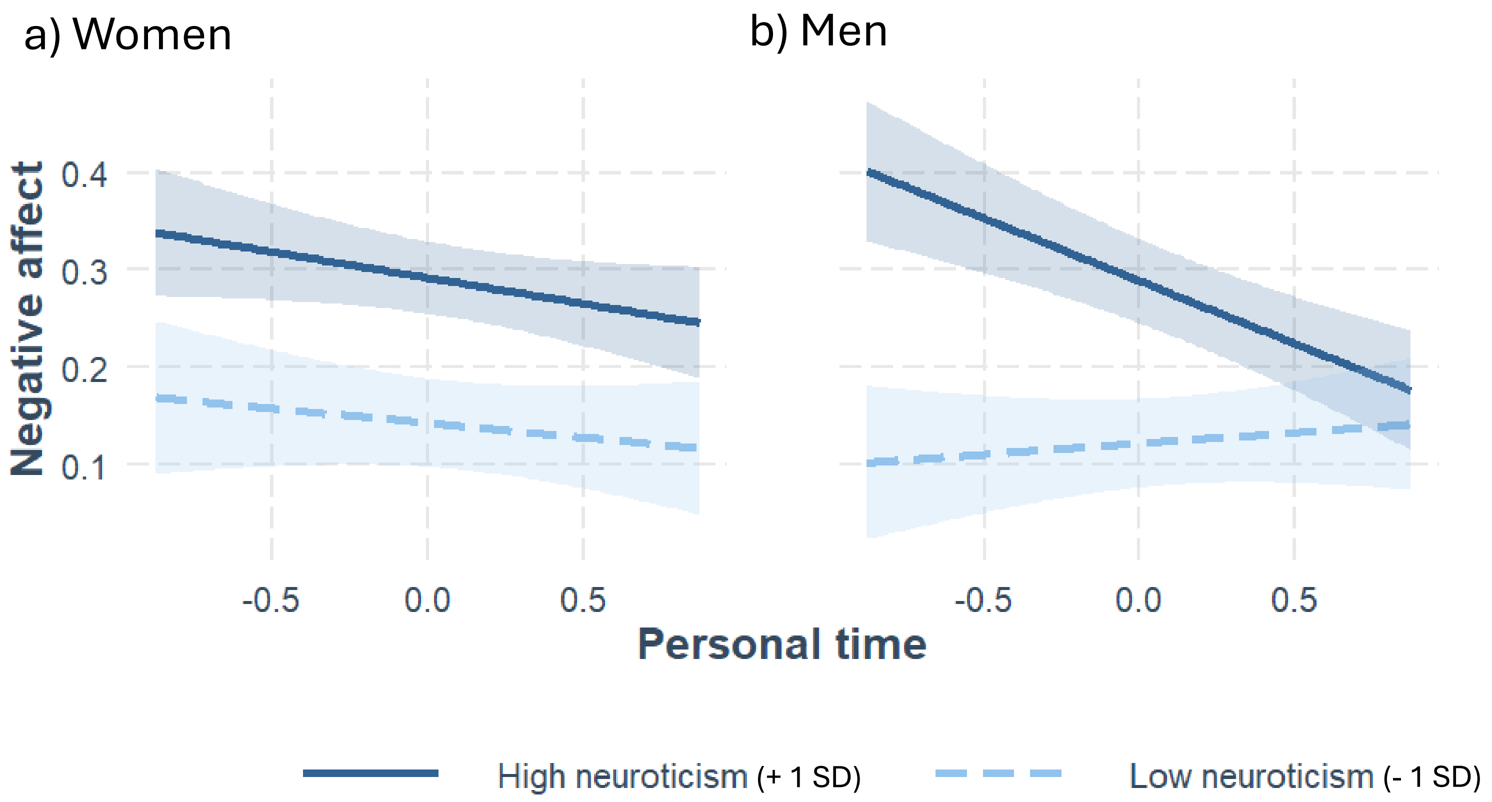

Note. The reduction in negative affect on days they had time to themselves was even greater for men (panel b) than for women (panel a) high in neuroticism, compared to those low in neuroticism. The shaded areas represents the uncertainty around each fitted line, calculated as fit ± standard error. Estimates are based on n = 1235 observations of N = 174 individuals for panel a and on n = 1064 observations of N = 144 individuals for panel b.
